# Adrenocortical oncocytic carcinoma with recurrent metastases: a case report and review of the literature

**DOI:** 10.1186/1477-7819-6-134

**Published:** 2008-12-17

**Authors:** Pinelopi Argyriou, Charalambos Zisis, Nektarios Alevizopoulos, Emmanuel M Kefaloyannis, Constantine Gennatas, Constantina D Petraki

**Affiliations:** 1 Department of Pathology, Evangelismos General Hospital, Ipsilantou Str., Athens, Greece; 2 Department of Thoracic and Vascular Surgery, Evangelismos General Hospital, Ipsilantou Str., Athens, Greece; 3 Oncology Clinic, Evangelismos General Hospital, Ipsilantou Str., Athens, Greece; 4 Oncology Clinic, Areteion Hospital, University of Athens, Vas. Sofias Av., Athens-Greece

## Abstract

**Background:**

Adrenal cortex oncocytic carcinoma (AOC) represents an exceptional pathological entity, since only 22 cases have been documented in the literature so far.

**Case presentation:**

Our case concerns a 54-year-old man with past medical history of right adrenal excision with partial hepatectomy, due to an adrenocortical carcinoma. The patient was admitted in our hospital to undergo surgical resection of a left lung mass newly detected on chest Computed Tomography scan. The histological and immunohistochemical study revealed a metastatic AOC. Although the patient was given mitotane orally in adjuvant basis, he experienced relapse with multiple metastases in the thorax twice in the next year and was treated with consecutive resections. Two and a half years later, a right hip joint metastasis was found and concurrent chemoradiation was given. Finally, approximately five years post disease onset, the patient died due to massive metastatic disease. A thorough review of AOC and particularly all diagnostic difficulties are extensively stated.

**Conclusion:**

Histological classification of adrenocortical oncocytic tumours has been so far a matter of debate. There is no officially established histological scoring system regarding these rare neoplasms and therefore many diagnostic difficulties occur for pathologists.

## Background

Hamperl introduced the term "oncocyte" in 1931 referring to a cell with abundant, granular, eosinophilic cytoplasm [1]. Electron microscopic studies revealed that this granularity was due to mitochondria accumulation in the oncocyte cytoplasm [2]. Neoplasms composed predominantly or exclusively of this kind of cells are called "oncocytic" [2]. Such tumours have been described in the overwhelming majority of organs: kidney, thyroid and pituitary gland, salivary, adrenal, parathyroid and lacrimal glands, paraganglia, respiratory tract, paranasal sinuses and pleura, liver, pancreatobiliary system, stomach, colon and rectum, central nervous system, female and male genital tracts, skin and soft tissues [2-15]. Adrenocortical oncocytic neoplasms (AONs) represent unusual lesions and three histological categories are included: oncocytoma (AO), oncocytic neoplasm of uncertain malignant potential (AONUMP) and oncocytic carcinoma (AOC) [3]. In our study, we add to the 22 cases found in the literature a new AOC with peculiar clinical presentation [16-29].

## Case presentation

**Figure 1:**
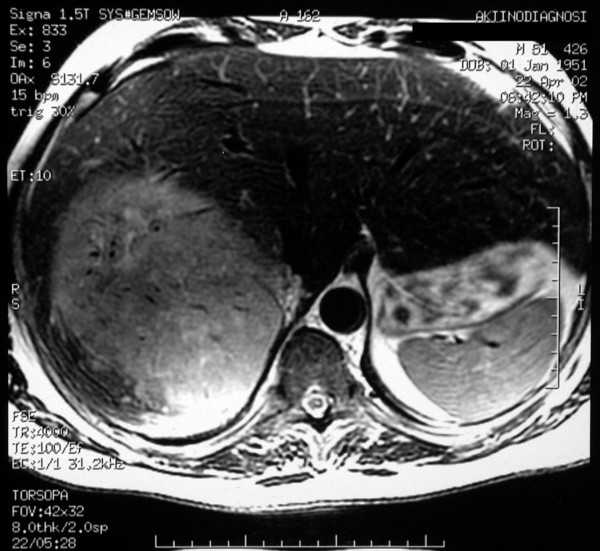
**Abdominal MRI showing the hepatic invasion, which was submitted to en block resection with the right adrenal**.

A 54-year-old man was admitted in the Thoracic and Vascular Surgery Department of our hospital with a 2 cm mass at the upper lobe of the left lung detected on Computed Tomography (CT) scan to undergo complete surgical resection. He had a past medical history of adrenocortical carcinoma (AC) treated surgically with right adrenalectomy and partial hepatectomy en block 2 years ago (Figure 1). He was a mild 3 pack year smoker and a moderate drinker (1/2 kgr wine/day).

**Figure 2:**
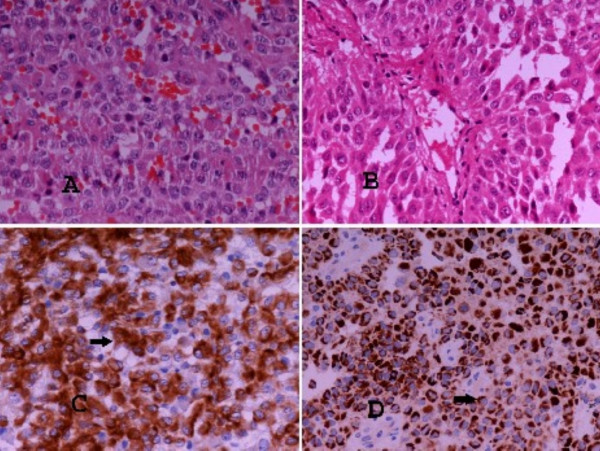
**A&B) Oncocytic adrenocortical carcinoma (A-H, magnification×200).** Diffuse (A) and papillary (B) histological pattern. **C) Vimentin immunohistochemical expression (magnification ×200). D) Melan-A immunohistochemical expression (magnification ×200).**

**Figure 3:**
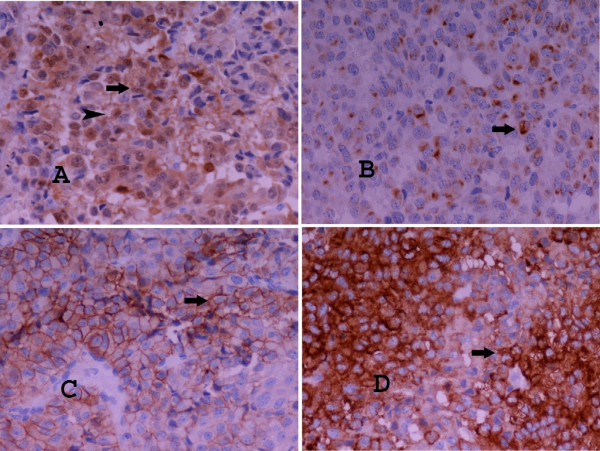
**A) Calretinin immunohistochemical expression (magnification ×200). **Fried-egg-like specific staining pattern.** B) CK8-18 immunohistochemical expression (magnification ×200). **Dot-like paranuclear expression. **C) CD56 immunohistochemical expression (magnification ×200). D) Synaptophysin immunohistochemical expression (magnification ×200).  **

Overall physical examination showed neither specific abnormality, nor any signs of endocrinopathy. All laboratory tests including cortisol, 17-ketosteroids and 17-hydrocorticosteroids serum levels and dexamethasone test, full blood count and complete biochemical hepatic plus renal function tests were in normal rates. The patient was subjected to wedge resection. Histological examination revealed a tumour with an oxyphilic cell population, moderate nuclear atypia, diffuse, rosette-like and papillary growth pattern and focal necroses (Figure 2a, b). A number of 4 mitotic figures/50 high power fields (HPFs) were documented. The proliferative index Ki-67 (MIB-1, 1:50, DAKO) was in a value range of 10–20% and p53 oncoprotein (DO-7, 1:20, DAKO) was weakly expressed in a few cells. Immunohistochemical examination revealed positivity for Vimentin (V9, 1:2000, DAKO), Melan-A (A103, 1:40, DAKO), Calretinin with a fried-egg-like specific staining pattern (Rabbit anti-human polyclonal antibody, 1:150, DAKO) and Synaptophysin (SY38, 1:20, DAKO). Both Cytokeratins CK8,18 (UCD/PR 10.11, 1:80, ZYMED) and AE1/AE3 (MNF116, 1:100, DAKO) showed a dot-like paranuclear expression. Inhibin-a (R1, 1:40, SEROTEC) and CD56 (123C3, 1:50, ZYMED) were expressed focally (Figures 2c, d and 3a–d). CK7 (OV-TL 12/30, 1:60, DAKO), CK20 (K_S _20.8, 1:20, DAKO), EMA (E29, 1:50, DAKO), CEAm (12-140-10, 1:50, NOVOCASTRA), CEAp (Rabbit anti-human polyclonal antibody, 1:4000, DAKO), TTF-1 (8G7G3/1 1:40, ZYMED), Chromogranin (DAK-A3, 1:20, DAKO) and S-100 (Rabbit anti-human polyclonal antibody, 1:1000, DAKO) were negative. Based on the morphological and immunohistochemical features of the neoplasm and the patient's past medical history, other oncocytic tumours were excluded and the diagnosis of a metastatic AOC was supported. Mitotane oral medication was given in adjuvant setting (2 g/d).

Seven months later, a new right lower lobe mass of 1.5 cm diameter was found on follow-up CT scan. A second wedge resection was performed including an excision of a nodule infiltrating the diaphragm. The histopathological examination confirmed diagnosis of AOC.

**Figure 4:**
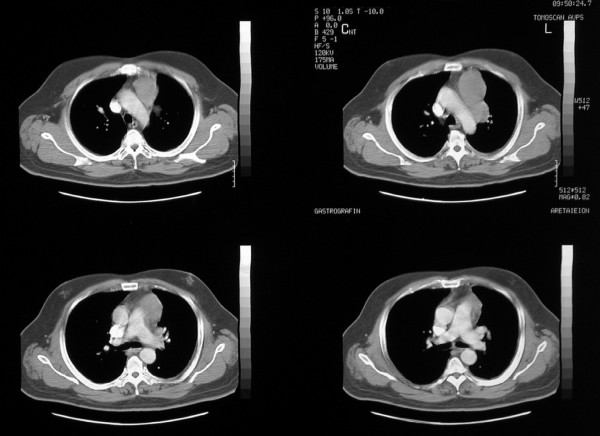
**Chest CT-scan revealing the sizeable mediastinal mass in the pre-aortic space extending into the aortopulmonary window**.

A new CT scan, six months later, demonstrated a lobulated mass, 2.8 cm in diameter, at the lingula and a lymph node block, measuring 11 × 5.5 cm at the preaortic space extending to the aortopulmonary window (Figure 4). The patient underwent a left upper lobectomy and radical mediastinal lymph node dissection. Histological examination confirmed AOC relapse with neoplastic spread around the superior lobar bronchus, invasion into branches of the pulmonary artery and metastatic infiltration of peribronchial and mediastinal lymph nodes.

**Figure 5:**
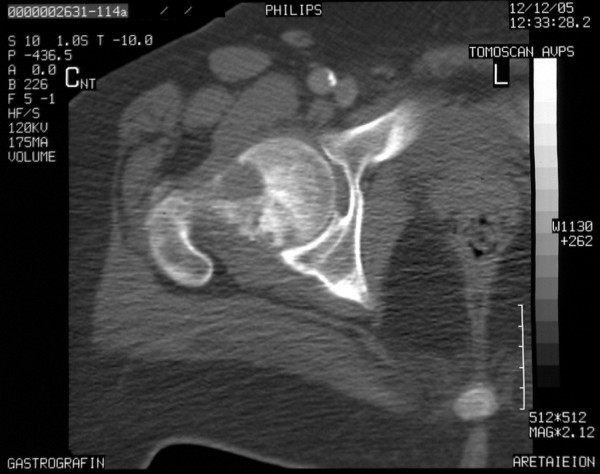
**Metastatic appearance of the right hip**.

Three and a half years post first surgery, a right hip joint metastasis was revealed on CT scan (Figure 5). The patient received three cycles of cisplatin based chemotherapy (75 mg/m^2 ^q 21 days) followed by three cycles of epirubicin (50 mg/m^2 ^q 21 days) and etoposide (100 mg/m^2 ^D1, D2, D3 q 21 days) combined chemotherapeutic regiments, concurrently with radiotherapy of right hip. No severe toxicity was stated. The therapeutic schedule combination with ongoing, orally given, mitotane was completed uneventfully. The patient remained in a good performance status (PS: 0) for 16 months and finally died, approximately 5 years post his disease onset, due to massive recurrence.

## Discussion

Adrenocortical tumours are usually solitary lesions and in their vast majority occur in adults without sex predilection [4]. Several histological systems have been proposed so far in a trial to predict the biological behaviour of these neoplasms [30-35]. Among them the Weiss system has the most important position and is widely used. This system supports that the presence of four or more of the following nine criteria (nuclear grade III-IV, mitotic rate >5/50 HPFs, atypical mitoses, clear cell tumour composition ≤ 25%, diffuse architecture, necrosis, venous, sinusoidal and capsular invasion) is indicative of malignancy. An increased number of mitoses, especially when combined with atypical forms, and venous invasion were best associated with malignancy [31]. The presence of more than 20 mitoses was correlated with more adverse clinical outcome and ACs with this criterion was suggested to be designated high grade [33].

Oncocytic variants of adrenocortical neoplasms are a special subgroup and whether the Weiss system can be used to evaluate their clinical behaviour is under consideration by several authors [18,22,24,25,36]. Lin et al believed that the assessment of AOCs should be conservative in the cases where mitotic activity, necrosis, capsular or vascular invasion are absent [36]. Furthermore, Krishnamurthy et al share similar opinion suggesting that the only unquestionable criterion of malignancy in an AON is the presence of metastasis or invasion (capsular and/or vascular) [18]. Hoang et al. added to the previous malignant features the presence of surgical unresectability and large tumour size [22]. Song et al. also agreed on the modification of the Weiss system [25].

More recently, Bisceglia et al. proposed new Weiss modified criteria and clearly determined the terms AO, AONUMP and AOC. More specifically, they suggested the following: a) if one of the criteria defined as major [high mitotic rate (>5 mitoses/50 HPF), atypical mitoses, venous invasion] is present in an AON, the latter should be considered malignant, b) if one to four of the criteria defined as minor [large size and/or huge weight (>10 cm and/or >200 gr), necrosis, capsular invasion, sinusoidal invasion] is found, the tumour should be deemed of uncertain malignant potential, and finally c) lack of both major and minor criteria indicates a benign lesion [24].

The role of the proliferative index (Ki-67) and oncoprotein p53 has also been a controversial issue in the past years. Some authors have suggested that these markers could be used as potential indicators of the benign or malignant nature of ACs [37-39]. Bisceglia et al results concerning Ki-67 expression of AONs were mostly in accordance with previous studies of the proliferative index in conventional ACs [24]. However, other authors studies showed that Ki-67 as long as p53 cannot be reliably used to predict the biological behaviour of AONs [18,22,25,36].

Literature review revealed 22 cases of AOC so far [16-29]. All data related to this histological subtype's clinical presentation, pathological fearures, outcome and therapeutic treatment approaches were studied. We tried to match them with our case data and furthermore to compare them to conventional ACs'. AOCs occur in adults between 25 and 77 years and no sex distribution is documented. In contrast with AOCs, ACs affect both children and adult population (range cited 43–67 years) and a female predilection is mentioned [40,41]. Histologically, AOCs differ from conventional ACs as they consist exclusively or predominantly of oncocytes; however the immunohistochemical profile of both neoplasms is similar. Patients with AOCs usually present with symptoms regarding abdominal mass and rarely regarding adrenal hormone imbalance production [16,17,22,23,25,26,28]. Furthermore, abnormal adrenal hormonal serum and urinary levels, without clinically suspected disease, have been noted in a few cases [23,24,26,27]. On the other hand, ACs usually present with high clinical evidence of adrenal hormonal hypersecretion (in 40–60% of cases) and less frequently with abdominal discomfort or back pain [41,42]. Literature data show that although invasion of other organs/structures beyond the primary tumour site and metastases may be found in both AOCs and ACs at the onset and/or later on, locally advanced disease does not occur in AOCs as often as in ACs on first diagnosis [16,19,22-27,29,41].

In our case, based on the exclusively oncocytic cell features of the neoplasm, a differential diagnosis among oncocytic tumours, either primary of the lung or metastatic, was needed. The patient's medical history and the neoplasm's immunohistochemical profile clarified its adrenocortical origin, its local infiltrative presentation and its malignant metastatic behaviour.

There is a wide discussion about the multimodality therapeutic approach which is needed apart from the radical surgical excision of the primary AC tumour, its local recurrences and relevant metastatic involved sites [42,43]. Radiotherapy, in adjuvant or symptomatic control setting seems to be delivered helpfully or as a standard care of palliation [42]. In clinical trials, metastatic AC extensive disease is treated with mitotane and multiple chemotherapeutic regiments combination (i.e. etoposide, doxorubicin, cisplatin or streptozotocin). Chemotherapy in adjuvant setting is under discussion so far [43]. The mainstay therapeutic approach in both ACs and AOCs is wide surgical resection. In AOCs, radiotherapy, mitotane and/or chemotherapy is given individually post bulky cytomassive excision, depending on disease staging and predominant symptoms. Our patient was treated according to the multimodality therapeutic combination.

It is the first time, in our knowledge, that an AOC was submitted to consecutive resections due to metastatic infiltration of both lungs and mediastinal lymph nodes, as if it was a primary lung cancer. In our case, neoplasm spreading may originate in carcinomatous emboli that entered the inferior vena cava; however, lymph node invasion has not been previously described at such a distant site. It is noteworthy that although this neoplasm had aggressive behaviour with constant relapse, the patient's performance status remained well. This fact dictated the aggressive surgical practice.

## Conclusion

Histological classification of adrenocortical oncocytic tumours has been so far a matter of debate. There is no officially established histological scoring system regarding these rare neoplasms and therefore many diagnostic difficulties occur for pathologists. Metastatic disease is the only definite criterion of malignancy. Molecular biology and large clinical studies may probably provide in the future more precise criteria for the classification, clinical behaviour and therapeutic approach of AOCs.

## Abbreviations

AC: Adrenocortical Carcinoma; AO: Adrenocortical Oncocytoma; AOC: Adrenocortical Oncocytic Carcinoma; AON: Adrenocortical Oncocytic Neoplasm; AONUMP: Adrenocortical Oncocytic Neoplasm of Uncertain Malignant Potential; CT scan: Computed Tomography scan; HPF: High Power Field.

## Consent

Written informed consent was obtained from the patient's relatives for publication of this case report and the accompanying images. A copy of the written consent is available for review by the Editor- in-Chief of this journal.

## Competing interests

The authors declare that they have no competing interests.

## Authors' contributions

PA did the macroscopic and microscopic examination of the specimens, collected and reviewed the literature data, prepared the figures, drafted, wrote, typed, formatted and revised the manuscript. CDP did the macroscopic and microscopic examination of the specimens, put the diagnosis, made the design, revised and supervised the manuscript. CZ operated on the patient, participated in the literature review and drafted the case presentation apart from the histopathological part. NA elaborated and revised the manuscript critically for stylistic imperfections, participated in the literature review and wrote the part of the manuscript regarding therapy approach. EMK operated on the patient, collected clinical data and participated in the literature review. CG was the attending oncologist, provided relevant clinical information and participated in the literature review.
